# Supplementary material for: Parapneumonic effusions related to Streptococcus pneumoniae: serotype and disease severity trends from 2006 to 2018 in Bristol, UK
Source: BMJ Open Respir Res. 2023 May 5;10(1):e001440. doi: 10.1136/bmjresp-2022-001440 (PMC10163460; doi:10.1136/bmjresp-2022-001440)
Supplement: Supplementary data [file bmjresp-2022-001440supp001.pdf]

Supplementary Data 1: Serotypes contained within each pneumococcal vaccination and vaccination group.

**Vaccination**	**Serotypes**
Pneumococcal polysaccharide vaccine, 23 valent (PPV-23, PneumoVax®)	1, 2, 3, 4, 5, 6B, 7F, 8, 9N, 9V, 10A, 11A, 12F, 14, 15B, 17F, 18C, 19F, 19A, 20, 22F, 23F, 33F.
Pneumococcal conjugate vaccine, 7-valent (PCV-7, Prevenar®)	4, 6B, 9V, 14, 18C, 19F and 23F
Pneumococcal conjugate vaccine, 13-valent (PCV-13, Prevenar13®)	1, 3, 4, 5, 6A, 6B, 7F, 9V, 14, 18C, 19A, 19F, and 23F
PCV13-7 serotypes	1, 3, 5, 6A, 7F, 19A

Supplementary Data 2: Study flow diagram

Results from 3677 microbiology database occurrences of a positive blood or urine antigen test were identified. There were 2657 cases of pneumococcal infection in adults, including 559 patients with parapneumonic effusions.

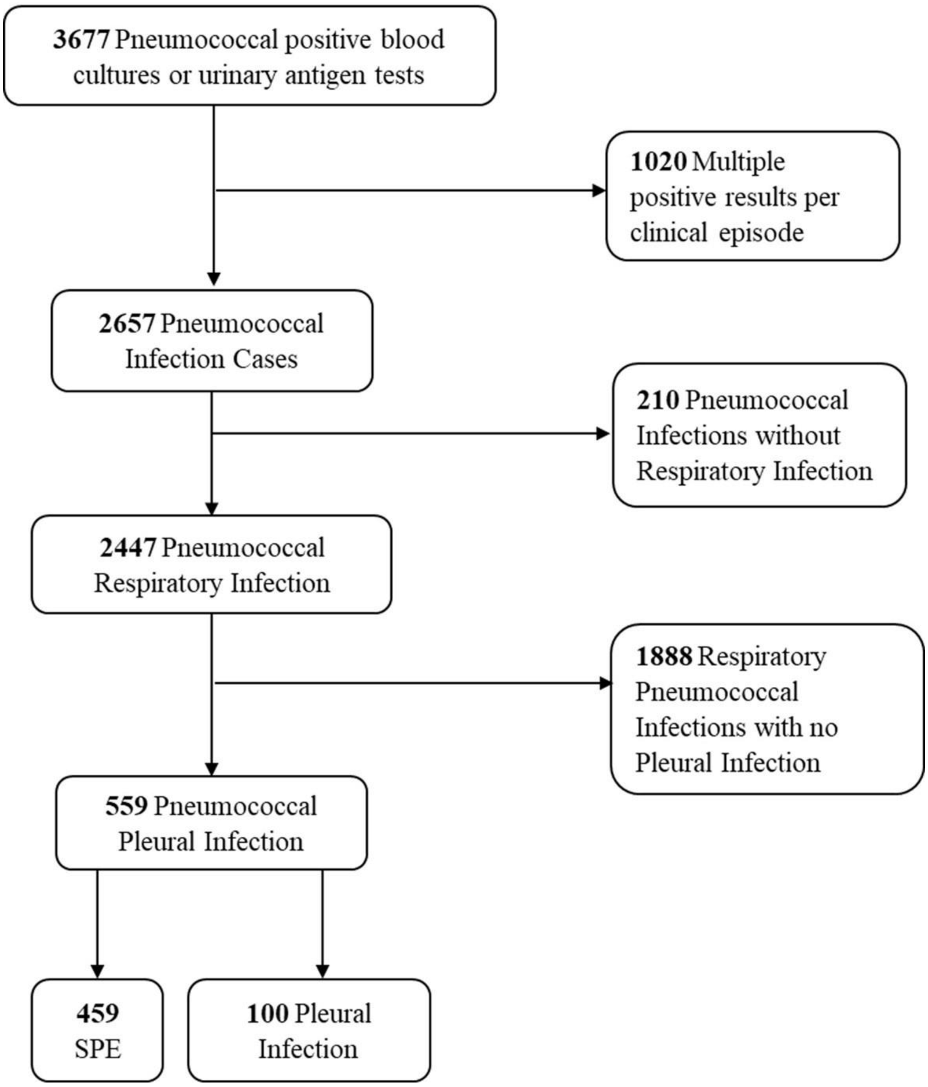

Supplementary Data 3: Additional characteristics and outcomes of patients admitted with pneumococcal parapneumonic effusions

Variable	Characteristic	SPE	Pleural infection	*P* value *
		N=459	N=100	
Index of Multiple Deprivation‡	Median [IQR]	6 [4—10]	6 [4—8]	—
**Cardiac Disease**				
Hypertension	Rate % (N)	46.2% (212)	34.0% (34)	0.027
Ischaemic Heart Disease	Rate % (N)	23.5% (108)	20.0% (20)	0.51
Atrial Fibrillation	Rate % (N)	18.1% (83)	11.0% (11)	0.1
Left Ventricular Failure	Rate % (N)	11.5% (53)	3.0% (3)	0.009
**Respiratory Disease**				
COPD	Rate % (N)	30.7% (141)	24.0% (24)	0.23
Asthma	Rate % (N)	9.6% (44)	12.0% (12)	0.46
**Malignancy and Immunosuppressive Conditions**				
Solid Organ Cancer	Rate % (N)	15.5% (71)	11.0% (11)	0.28
Haematological Malignancy	Rate % (N)	9.6% (44)	8.0% (8)	0.71
Autoimmune Disease	Rate % (N)	7.6% (35)	7.0% (7)	1
Chemotherapy	Rate % (N)	3.9% (18)	2.0% (2)	0.55
Corticosteroid use	Rate % (N)	4.1% (19)	3.0% (3)	0.78
On Immunosuppressants	Rate % (N)	4.4% (20)	1.0% (1)	0.15
**Vaccination status #**				
PPV23 Vaccination	None % (N)	51.4% (236)	65.0% (65)	0.0028
	Under 6 months % (N)	4.4% (20)	8.0% (8)	
	Over 6 months % (N)	44.2% (203)	27.0% (27)	
Influenza Vaccination	None % (N)	48.1% (221)	54.0% (54)	0.34
	Under 6 months % (N)	42.7% (196)	35.0% (35)	
	Over 6 months % (N)	9.2% (42)	11.0% (11)	
**Admission Observations and Clinical Features**				
Respiratory rate (min^{-1})	Median [IQR]	28 [24—32]	30 [24—34]	0.12
Oxygen Sats (%)	Median [IQR]	88 [84—91]	86 [83—90]	—
Confusion	Rate % (N)	28.8% (132)	30.0% (30)	0.81
Hypotensive at admission	Rate % (N)	14.8% (68)	20.0% (20)	0.22
**Treatment**				
Non-invasive ventilation	Rate % (N)	7.6% (35)	2.0% (2)	0.044
Endotracheal intubation	Rate % (N)	17.6% (81)	11.0% (11)	0.14
Tracheostomy	Rate % (N)	4.4% (20)	6.0% (6)	0.44
Inotropes required	Rate % (N)	17.4% (80)	9.0% (9)	0.035

* Significance determined using Fisher's exact test (categorical variables) or not calculated due to missing values, 2 sample Wilcoxon Rank Sum test (continuous variables). Normality of distributions determined using Anderson-Darling normality test

‡ Index of deprivation was calculated using postcode to derive Lower-layer Super Output Area (LSOA) data from The Office of National Statistics (ONS)

# Vaccination status for pneumococcal and seasonal influenza was verified for each patient from GP records.

Supplementary Table 4: Annual population and hospital admissions

Population size within the Bristol, North Somerset and South Gloucestershire CCG and Bath & NE Somerset CCG as based on data obtained from the Office for National Statistics (ONS). Number of adult patients seen are listed as a combined total for all 3 NHS Trusts, from data provided by NHS Digital. Data for patients seen in Emergency Departments is derived from Hospital Accident and Emergency Activity. Data for admissions is collated from Monthly Hospital Episode Statistics for Admitted Patient Care, Outpatient and Accident and Emergency data

**Characteristic – N**	2006	2007	2008	2009	2010	2011	2012	2013	2014	2015	2016	2017	2018
**Population**													
Whole population	1,031,918	1,040,674	1,047,210	1,053,791	1,028,596	1,070,120	1,080,886	1,131,336	1,104,211	1,118,820	1,131,336	1,139,791	1,140,236
≥ 16 y	823,298	833,548	839,310	844,186	866,522	887,470	925,556	893,556	902,950	915,438	925,566	933,304	940,293
16-34 y	237,508	287,368	288,798	288,536	297,243	306,361	301,317	301,317	305,932	313,489	319,384	322,914	323,715
35-49 y	222,704	224,970	225,290	225,944	227,259	220,217	219,883	219,883	217,895	217,136	216,015	215,618	215,987
50-64 y	176,998	178,690	179,822	180,714	184,550	186,294	185,960	185,960	188,442	191,215	194,082	196,991	197,565
65-79 y	106,488	107,636	109,918	112,516	118,948	129,021	140,629	133,018	136,428	138,803	140,629	141,792	142,895
≥80 y	34,094	34,884	35,487	36,476	38,522	45,577	55,456	53,378	54,253	54,795	55,456	55,989	60,131
**Adults Admissions**													
Accident & Emergency	266,411	261,328	270,012	266,714	268,253	271,948	279,953	286,314	275,960	28,3137	263,566	269,868	271,328
Unplanned admissions	103,590	96,665	123,004	105,774	108,151	107,704	106,753	109,235	114,415	120,960	126,315	133,640	137,521
RR admissions *	1.00	0.93	1.19	1.02	1.04	1.04	1.03	1.05	1.10	1.17	1.22	1.29	1.33
**Tests Conducted**													
Blood Cultures	41,975	42,586	42,033	43,735	44,983	46,620	46,021	47,955	48,336	50,028	50,852	51,186	52,904
Urine Antigen	52	143	308	960	1,591	1,909	2,141	2,251	2,645	3,517	3,535	3,706	3,405
RR testing *	1.00	1.02	1.01	1.06	1.11	1.15	1.15	1.19	1.21	1.27	1.29	1.31	1.34

* The relative ratio of unplanned hospital admissions and microbiological testing (blood culture and urine antigen combined) were calculated using 2006 as the reference year

Supplementary Data 5: Box plot of age of adults hospitalised with parapneumonic effusions and all medical admissions 2006-2018

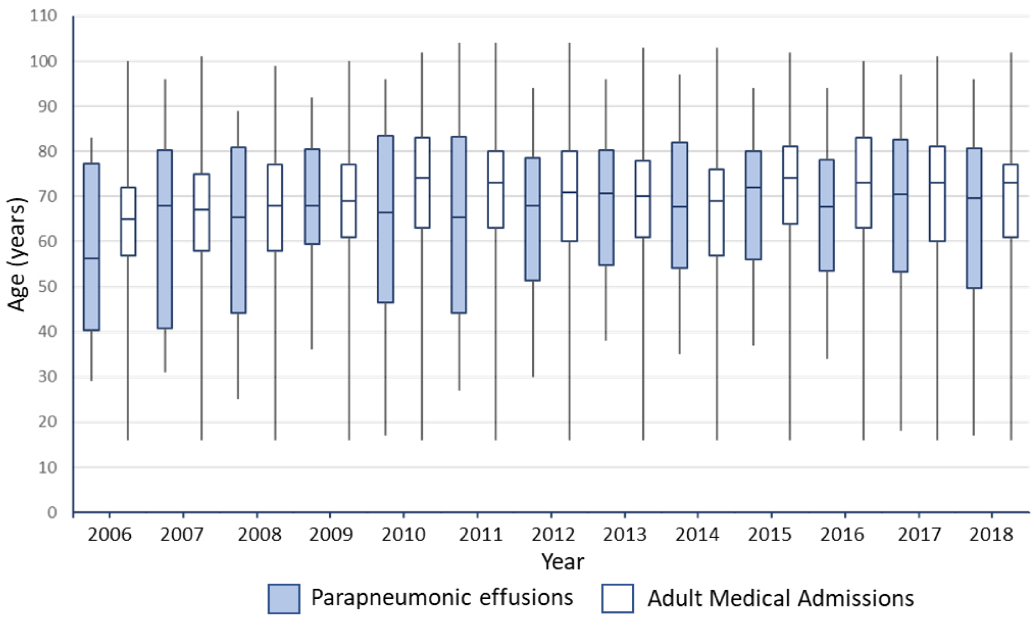

Supplementary Data 6: Pleural disease attributable to significant serotypes throughout the study

The percentage of pleural disease caused by significant serotypes from (A) PCV7, (B) PCV13-7 and (C) non-vaccine serotype groups, as a percentage of the total known serotype disease caused within year group

(A) PCV7 serotypes

Year	4	6B	14	19F	23F
2006-2008	2.5	7.5	25.0	2.5	5.0
2009-2011	2.7	2.7	2.7	1.3	4.0
2012-2014	0.0	0.0	2.2	2.2	0.0
2015-2018	0.0	0.0	1.6	0.0	0.0

(B) PCV13-7 serotypes

Year	1	3	6A	7F	19A
2006-2008	12.5	10.0	10.0	0.0	5.0
2009-2011	13.3	13.3	1.3	12.0	10.7
2012-2014	6.5	13.0	0.0	0.0	0.0
2015-2018	6.6	16.4	0.0	0.0	0.0

(C) Non-vaccine serotypes

Year	8	12F	22F
2006-2008	0.0	2.5	2.5
2009-2011	4.0	4.0	6.7
2012-2014	8.7	8.7	10.9
2015-2018	16.4	14.8	9.8

Supplementary Data 7: Characteristics of Patients with pleural infection, by survival following presentation

Characteristic - N (%)	**All Parapneumonic effusions**		**SPE**		**Pleural Infection**	
	Survived >90 days	Died within 90 days	Survived >90 days	Died within 90 days	Survived >90 days	Died within 90 days
	N=453	N = 106	N=365	N = 94	N = 86	N = 14
Gender						
- Male	228 (50)	57 (56)	181 (49)	50 (53)	45 (52)	7 (50)
- Female	225 (50)	49 (46)	184 (51)	44 (47)	41 (47)	7 (50)
Age – mean (IQR)	69 (52-89)	78 (68-84)	68 (50-80)	80 (68-89)	56 (44-71)	73 (67-88)
Smoking Status						
- Current smoker	174 (38)	28 (26)	128 (35)	26 (28)	44 (51)	3 (21)
- Ex smoker	180 (40)	61 (58)	159 (43)	52 (55)	21 (24)	9 (64)
Vaccination Status						
- PneumoVax (PPV23)	204 (45)	56 (58)	176 (48)	47 (50)	27 (32)	9 (64)
- Seasonal Flu	239 (53)	39 (40)	195 (53)	37 (39)	43 (50)	5 (35)
Pneumococcal vaccine serotype						
Unknown serotype	282/453 (62)	55/106 (52)	231/365 (63)	50/94 (53)	50/86 (58)	5/14 (35)
PCV13 vaccine serotype	95/181 (52)	31/57 (54)	65/143 (45)	26/50 (52)	30/36 (83)	5/9 (56)
Non-vaccine serotype (NVT)	16/181 (8)	6/57 (11)	14/143 (10)	5/50 (10)	2/36 (5)	1/9 (11)
CPE/Empyema features						
- Pus	59 (13)	0 (0)	-	-	59 (66)	0 (0)
- Loculation	39 (9)	10 (10)	-	-	39 (45)	10 (71)
- Fibrinolytic treatment	27 (6)	6 (6)	0 (0)	0 (0)	27 (31)	6 (43)
- Surgical Management	29 (6)	0 (0)	0 (0)	0 (0)	29 (34)	0 (0)
Co-morbidities						
Chronic Resp Disease	166 (37)	54 (51)	130 (36)	49 (52)	36 (42)	5 (36)
COPD	123 (27)	44 (42)	104 (28)	40 (42)	19 (22)	4 (29)
Asthma	48 (11)	9 (8)	35 (9)	9 (10)	13 (15)	0 (0)
Other Chronic Resp Disease	57 (10)	11 (10)	50 (13)	9 (10)	7 (8)	2 (14)
Chronic Cardiac Disease	226 (50)	74 (69)	190 (52)	68 (72)	36 (42)	6 (43)
Hypertension	187 (41)	57 (54)	156 (43)	59 (63)	31 (36)	4 (33)
Ischaemic Heart Disease	89 (19)	38 (36)	71 (19)	39 (41)	18 (21)	1 (7)
AF	63 (14)	27 (25)	59 (16)	23 (24)	4 (5)	4 (29)
LVF	42 (9)	13 (12)	39 (11)	13 (14)	3 (3)	0 (0)
Other CVS	16 (4)	2 (2)	16 (4)	5 (5)	0 (0)	2 (14)
Immunosuppression						
Autoimmune Disease	24 (5)	8 (8)	16 (4)	7 (7)	8 (9)	1 (7)
Oral Corticosteroids	18 (4)	4 (4)	16 (4)	3 (3)	2 (2)	1 (7)
Other immunosuppressive med	17 (4)	6 (6)	14 (4)	5 (5)	3 (3)	1 (7)
Solid organ cancer	58 (13)	24 (23)	49 (13)	21 (21)	9 (10)	3 (21)
Haematological malignancy	38 (8)	13 (12)	34 (9)	10 (11)	4 (5)	3 (21)
Recent chemotherapy	15 (3)	5 (5)	14 (4)	4 (4)	1 (1)	1 (7)
Other Risk Factors						
Upper GI/Swallow problem	168 (37)	54 (52)	136 (37)	48 (51)	29 (34)	6 (43)
Chronic Liver Disease	17 (4)	8 (8)	14 (4)	7 (7)	3 (3)	1 (7)
Diabetes Mellitus	81 (18)	16 (15)	66 (18)	16 (17)	15 (17)	0 (0)
CVA disease	24 (5)	13 (12)	19 (5)	10 (11)	5 (6)	3 (21)

Alcohol misuse	41 (9)	11 (10)	29 (8)	9 (10)	12 (14)	2 (14)
Intravenous drug usage	16 (3)	8 (8)	10 (3)	7 (7)	6 (7)	1 (7)
Obesity or BMI<17	16 (3)	4 (4)	9 (3)	4 (4)	7 (8)	0 (0)
Admission Features						
Length of Stay – median (IQR)	11 (7-23)	14 (6-24)	10 (5-19)	10 (4-21)	17 (11-24)	18 (11-28)
ICU/HDU care	93 (20)	20 (20)	75 (20)	18 (21)	18 (21)	2 (14)
- Intubation and ventilation	73 (16)	20 (20)	62 (17)	18 (21)	11 (13)	2 (14)
- Inotropic support	71 (15)	21 (21)	62 (17)	19 (22)	9 (10)	2 (14)

Supplementary Data 8: Testing of proportional hazards in Cox Regression model

(A) Log-Log(survival) curve plot of SPE (red line) and pleural infection (blue line) cases in the cohort. Scaled Schoenfeld residuals are shown for (B) age and (C) loculation, demonstrating log hazard-ratio function is constant over time as gradient of each slope is zero.

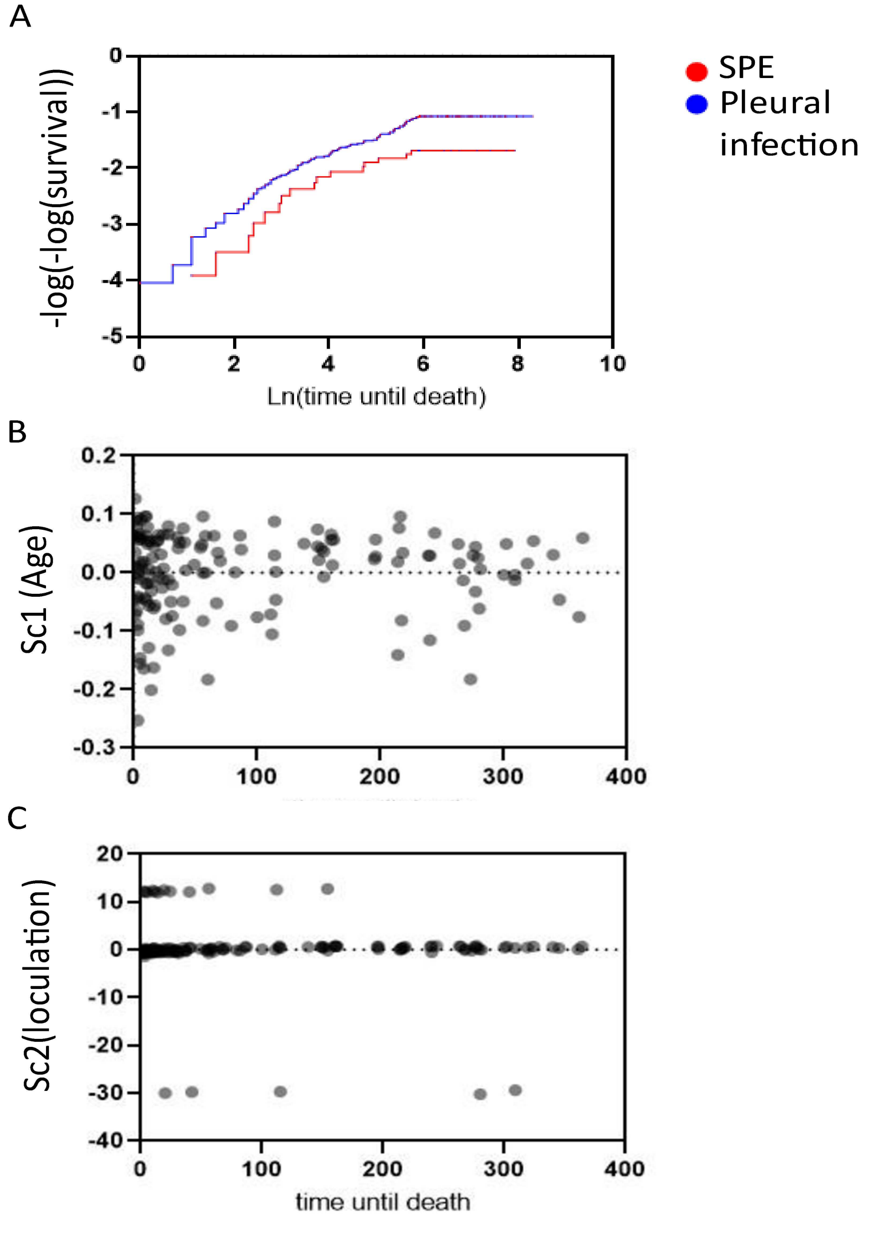

Supplementary Data 9: Table of Abbreviations and Definitions

Abbreviation	Explanation
AF	Atrial Fibrillation
AMTS	Abbreviated Mental Test Score
BMI	Body Mass Index
CAP	Community Acquired Pneumonia
COPD	Chronic Obstructive Pulmonary Disease
CPE	Complex Parapneumonic Effusion
CVA	Cerebrovascular Accident
CVS	Cardiovascular System
ETT	EndoTracheal Tube
HIV	Human Immunodeficiency Virus
HDU	High dependency unit
IHD	Ischaemic Heart Disease
IPD	Invasive Pneumococcal Disease
IQR	Interquartile Range
ITU	Intensive Therapy Unit
LRTI	Lower Respiratory Tract Infection
LSOA	Lower-layer Super Output Area
LVF	Left Ventricular Failure
MALDI-TOF	Matrix-assisted laser desorption/ionisation/time of flight
ONS	The Office of National Statistics
PCV	Polysaccharide conjugate vaccine
PHE	Public Health England
PPV23	Pneumococcal Polysaccharide Vaccine
RAPID	Renal, Age, Purulence, Infection source, & Dietary factors Score
SD	Standard Deviation
SPE	Simple Parapneumonic Effusion
ST	Serotype
